# Oral isoflavone supplementation on endometrial thickness: a meta-analysis of randomized placebo-controlled trials

**DOI:** 10.18632/oncotarget.7959

**Published:** 2016-03-07

**Authors:** Jie Liu, Feixiang Yuan, Jian Gao, Boer Shan, Yulan Ren, Huaying Wang, Ying Gao

**Affiliations:** ^1^ Key Laboratory of Nutrition and Metabolism, Institute for Nutritional Sciences, Shanghai Institutes for Biological Sciences, Chinese Academy of Sciences, Shanghai, China; ^2^ Nutrition Department, Affiliated Zhongshan Hospital of Fudan University, Shanghai, China; ^3^ Department of Female Tumor, Fudan University Shanghai Cancer Center, Shanghai, China

**Keywords:** isoflavone supplementation, endometrial thickness, meta-analysis, randomized controlled trials, Gerotarget

## Abstract

**Background:**

Isoflavone from soy and other plants modulate hormonal effects in women, and the hormone disorder might result in different caners including endometrial cancer. However, it's effect on the risk of endometrial cancer is still inconclusive. We aimed to assess the effects of isoflavone on endometrial thickness, a risk factor of endometrial cancer in peri- and post-menopausal women.

**Methods:**

A meta-analysis of randomized controlled trials was conducted to evaluate the effect of oral isoflavone supplementation on endometrial thickness in peri- and post-menopausal women. Electronic searches were performed on the PubMed, Embase, the Cochrane Library, web of science, CINAHL, and WHO ICTRP to August 1^st^, 2015. Reviews and reference lists of relevant articles were also searched to identify more studies. Summary estimates of standard mean differences (SMD's) and 95%CIs were obtained with random-effects models. Heterogeneity was evaluated with meta-regression and stratified analyses.

**Results:**

A total of 23 trials were included in the current analysis. The overall results did not show significant change of endometrial thickness after oral isoflavone supplementation (23 studies, 2167subjects; SMD:-0.05; 95%CI:-0.23, 0.13; *P*=0.60). Stratified analysis suggested that a daily dose of more than 54mg could decrease the endometrial thickness for 0.26mm (10 trials, 984subjects; SMD:-0.26; 95%CI:-0.45, −0.07; *P*=0.007). Furthermore, isoflavone supplementation significantly decrease the endometrial thickness for 0.23mm in North American studies (7 trials, 726 subjects; SMD:-0.23; 95%CI:-0.44, −0.01; *P*=0.04), but it suggested an increase for 0.23mm in Asian studies (3 trials, 224 subjects; SMD: 0.23; 95%CI:-0.04, 0.50; *P*=0.10).

**Conclusion:**

Oral isoflavone supplementation might have different effects in different populations and at different daily doses. Multiple-centre, larger, and long-term trials are deserved to further evaluate its effect.

## INTRODUCTION

In the endometrium, excess estrogen relative to progesterone produces a proliferative stimulus, which may result in endometrial thickening. As measured by transvaginal ultrasound (TVU), endometrial thickness can be a biomarker for the proliferative effects of estrogens, and opposing different influences of progesterone. The increase of endometrial thickness may be associated with increased risk of endometrial carcinomas [1, 2].

Phytoestrogens are plant-derived chemicals [3]. There is a large family of different classes of phytoestrogens, and isoflavones are the major type with highest activity that have been given therapeutically to women [4]. The structure of plant-derived isoflavone is similar to human 17β-estradiol. Isoflavones show selective estrogen receptor modulator like activity, though it's estrogenic and anti-estrogenic effects vary depending on the receptors of different target tissues [5]. This stimulated significant interest in the importance of isoflavone to women's health [6].

Isoflavone, mainly produced by soybeans, has been suggested to have estrogenic effects in human studies. Epidemiology studies suggested that dietary isoflavone could influence hormonal levels in women [7]. There have been many studies shown that given isoflavone product to postmenopausal women might relieve menopausal symptoms such as hot flushes and vaginal dryness [8, 9]. There are also studies evaluated the effect of oral isoflavone supplementation on endometrial thickness, a risk factor of endometrial cancer [10-32]. However, the results were not consistent, and the sample sizes were relatively small (vary from 15 to 401). Therefore, we searched all published, double-blinded, randomized and controlled trials, and conducted a meta-analysis to systematically evaluate the effect of isoflavone supplementation on endometrial thickness.

## RESULTS

### Search results

**Figure 1:**
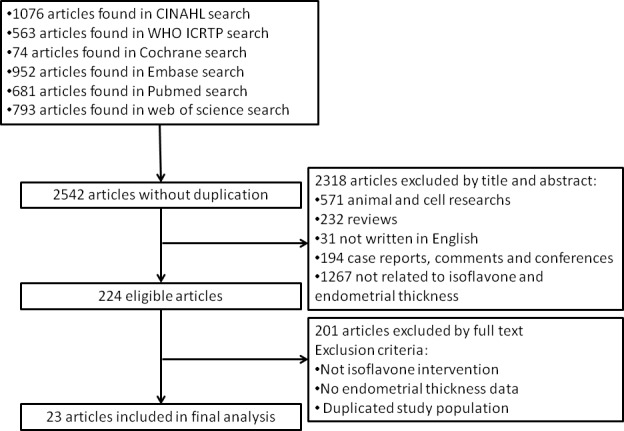
Identification process for eligible studies

The procedure of selection of studies is shown in Figure 1. In total, 2542 articles were identified in a combined search of the PubMed, Embase, Cochrane Library and web of science, CINAHL and WHO ICTRP databases, and reference lists of relevant articles (relevant text words “isoflavone” paired with “endometrial” or “endometrium” are used for searching). Of the 2542 articles, 2506 were excluded because they were animal experiments, *in vivo* experiments, or not relevant through abstract. After further excluded studies in which isoflavone intervention method was not appropriate, endometrial thickness measurements were not performed, endometrial thickness values were not reported, or the studies were not randomized placebo-controlled studies, 23 eligible randomized controlled studies [10-32] were finally left in the current meta-analysis.

### Description of the included studies

All of the included studies are randomized controlled trials (RCT). For a study with a crossover design, data from the first stage were extracted for the current study [25]. Total 2305 subjects from the 23 RCTs were included in the current analysis. The intervention duration lasted from 3 months to 3 years. The average age of the subjects ranged from 47 to 73 years. The details of characteristics of these studies are shown in Table 1.

**Table 1:** Characteristics of studies

Reference	Year	Study design	Participants	No. of subjects	Geographic regions	Baseline endometrial thickness^1^_T^2^ (mm)	Baseline endometrial thickness^1^_C^2^(mm)	Isoflavone dose (mg/d)	Source of isoflavone	Study duration (week)
D.H.Upmlis et al.	2000	Double-blind RCT	postmenopausal	122	USA	3.5±1.9	3.7±2.7	50	Soy	12
G. E. Hale et al.	2001	Double-blind RCT	Perimenopausal	24	Austria	7.09±2.85	6.46±1.7	50	Red clover	12
Kyung K.Han et al.	2002	Double-blind RCT	Perimenopausal	80	Brazil	3.3±0.1SE	2.6±0.1SE	100	Isoflavone tablet	16
A.Sammartino et al.	2003	RCT	postmenopausal	63	Europe	3.2(2.3-4.5)	3.1(2.3-4.2)	36	Genistein	48
M.J.Murray et al.	2003	RCT	postmenopausal	15	USA	3±1	3.6±1.6	120	Isoflavone tablet	24
M.Penotti et al.	2003	Double-blind RCT	postmenopausal	62	Europe	2.6±1.8	3.2±1.8	36	Soy	24
A.Crisafulli et al.	2004	Double-blind RCT	postmenopausal	60	Europe	3.2±0.25SE	3.3±0.33SE	54	Isoflavone tablet	48
E.Nikander et al.	2005	double blind cross over RCT	postmenopausal	56	Europe	2.4±1.5	2.1±1.1	114	Isoflavone tablet	12
M.Imhof et al.	2006	crossover RCT	postmenopausal	109	Austria	/	/	80	Red clover	13
E.A.P.Nahas et al.	2007	Double-blind RCT	postmenopausal	76	Brazil	3median(2.2-4.1) 25-75th	3.7median(2.6-4.1) 25-75th	100	Glycine max	40
G.Cheng et al.	2007	Double-blind prospective randomized study	postmenopausal	51	Europe	2.3±1.1	2±1	60	Soya beans	12
H.Marini et al.	2007	Double-blind RCT	postmenopausal	389	Italy	3.1±1.5	3.2±1.8	54	Isoflavone tablet	104
G.Zhang et al.	2007	Double-blind RCT	postmenopausal	100	China	1.82±0.62	1.75±0.58	18	Isoflavone tablet	12
J.Manonai et al.	2008	Double-blind RCT	postmenopausal	71	Asia	4.07±1.48	4.07±1.39	20,30,50	Pueraria mirifica	24
T.J.Powles et al.	2008	Double-blind RCT	postmenopausal	401	England	/	/	40	Red clover	156
A.M.Kenny et al.	2009	Double-blind RCT	postmenopausal	66	USA	2.97±0.81	3.14±0.81	57	Soy	52
R.D'Anna et al.	2009	RCT	postmenopausal	397	Europe	3.1±0.1SE	3.2±0.1SE	54	Isoflavone tablet	104
F.M.Steinberg et al.	2011	Double-blind RCT	postmenopausal	268	Multicenter	1.8±0.98	2±1.22	102.3	Synthetic	104
M.Evans et al.	2011	Double-blind RCT	postmenopausal	83	Canada	4.28±1.98	3.66±1.21	30	Synthetic	12
A.Oyama et al.	2012	Double-blind RCT	postmenopausal	68	Japan	0.2±0.8	0.1±0.5	5	Soy germ fermentation	12
A.M.Quaas et al.	2013	Double-blind RCT	postmenopausal	224	USA	2.4±1	2.5±1.1	154	Isolated soy protein	156
N.Colacurci et al.	2013	RCT	postmenopausal	124	Europe	3.35±0.95	3.47±1.07	60	Isoflavone tablet	48
D.L.Alekel et al.	2014	Double-blind RCT	postmenopausal	168	USA	1.7±1.1	1.3±0.65	120	Soy bean	156

1 All values are the means ±SDs, means ±SEs, mean (range), median (25th-75th range), or mean (95%CI) reported in the trial

2 T: treatment group, C: control group

Except two of the studies included peri-menopausal women [30, 31], all others only included post-menopausal women. Three studies provided red clover-based isoflavone supplements [18, 24, 31], one provided pueraria mirifica-based isoflavone supplements [19], seven provided additional soy foods [10, 13, 16, 21, 23, 28, 32], one provided soy protein powder [12], two provided synthetic isoflavone [14, 15], and the other nine provided soy-based isoflavone supplements [11, 17, 20, 22, 25-27, 29-30]. All the studies reported no side effect of supplementation in articles. Isoflavone supplementation doses ranged from 5 to 154mg/d and supplementation duration ranged from 12 to 156 weeks. Control groups received placebo and were advised to keep their usual diet. All studies evaluated endometrial thickness by transvaginal ultrasounds. The baseline endometrial thickness varied from 0.4±2.3mm to 7.25±2.88mm.

### Data quality

The quality of the 23 studies ranged from 3 to 5 scores (highest score). Exact details of randomization (mention of randomization methods, appropriateness of randomization), blinding (mention of blinding methods, appropriateness of blinding), and dropout (the fate of all patients in the trial was known) were reported in 14 studies [10, 12-15, 17, 20-23, 26, 28, 30-31], but not all mentioned in the other 9 studies [11, 16, 18-19, 24-25, 27, 29, 32].

### Effects of oral supplementation of isoflavone on endometrial thickness

The primary outcome of current study was the changes of the endometrial thickness compared to baseline after isoflavone supplementation. Three studies [12, 16, 18] reported the absolute changes of endometrial thickness, and the remaining 20 studies provided baseline and final endometrial thickness after intervention.

**Figure 2:**
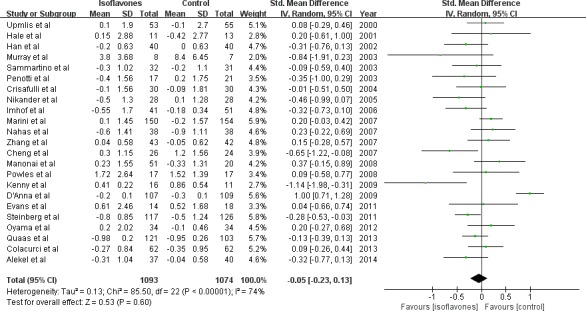
Meta-analysis of the effect of isoflavone supplementation on endometrial thickness The sizes of the data markers indicate the weight of each study in the analysis.

The overall results from pooling the 23 studies did not show significant change in endometrial thickness in either treatment or placebo groups (23 studies, 2167 subjects; SMD:-0.05; 95%CI:-0.23, 0.13; *P* = 0.60) (Figure 2). Significant heterogeneity for the outcome was found (Chi^2^ = 87.23, I^2^ = 74%, *P* < 0.00001). To detect the source of the heterogeneity, meta-regression and stratified analysis were performed. Meta-regression analysis showed that geographic region of the subjects (regression coefficient = −1.17; 95%CI: −1.97, −0.37 *P* = 0.004) and daily dose of isoflavone supplementation were negatively related to the effect size of heterogeneity (regression coefficient = −0.008; 95%CI: −0.19, −0.02 *P* = 0.05). The source of isoflavone (results not shown) and total dose of isoflavone supplementation did not modify the effect substantially according to meta-regression.

**Figure 3:**
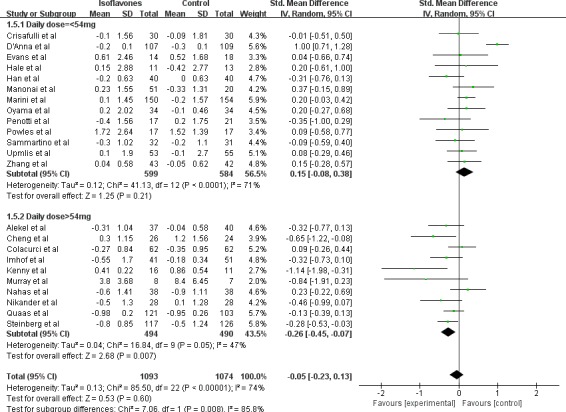
Meta-analysis of subgroup by geographic regions of study objects The sizes of the data markers indicate the weight of each study in the analysis. The geographic region is differentiated by study geographic region reported by trials.

To explore the modification effect, we performed stratified analysis according to median age, BMI, menopausal state, geographic regions, isoflavone total dose, and isoflavone daily dose (Table 2). We found that a significant decrease of endometrial thickness (−0.23mm) after isoflavone supplementation intervention (7 trials, 726 subjects; SMD:-0.23; 95%CI:-0.44, −0.01; *P* = 0.04) in subjects from North America. This was opposite to an increased change (0.23mm) observed in Asian subjects (3 trials, 224 subjects; SMD: 0.23; 95%CI:-0.04, 0.50; *P* = 0.10) (Figure 2). When daily isoflavone supplementation dose was more than 54mg the endometrial thickness was decreased (−0.26mm) significantly (10 trials, 984subjects; SMD:-0.26; 95%CI:-0.45, −0.07; *P* = 0.007) but the change was not significant (0.15mm) when the dose was less than 54mg (13 trials, 1183 subjects; SMD: 0.15; 95%CI:-0.08, 0.38; *P* = 0.21) (Figure 3). The study subjects of daily dose more than 54mg were all post-menopausal women. No significant modification effect was observed for median age, BMI, menopausal state, and isoflavone total dose.

**Figure 4:**
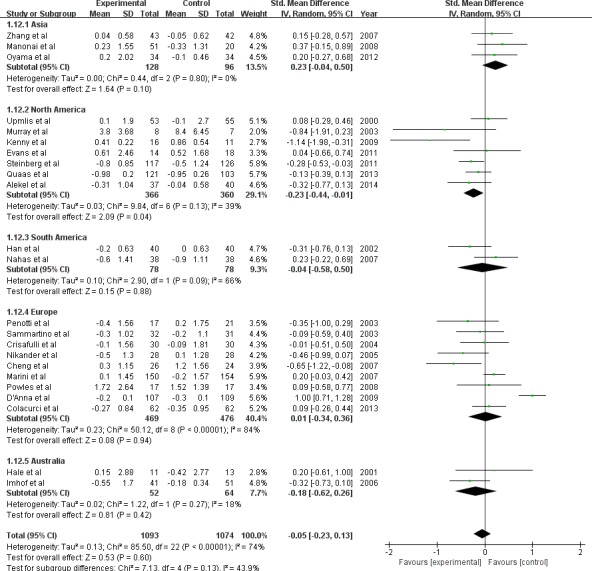
Meta-analysis of subgroup by dose of daily isoflavone supplementation The sizes of the data markers indicate the weight of each study in the analysis. The daily dose is based on the data counted by trials.

**Table 2:** Subgroup analyses for the effect of oral isoflavone supplementation on endothelial thickness

	Study number	Number of subjects	SMD^1^ (95%CI)	P^2^
Age				
≤60y	20	1931	−0.02(−0.22,0.18)	0.84
>60y	3	336	−0.24(−0.75,0.26)	
Daily Isoflavone dose				
≤54mg/d	13	1183	0.15(−0.08,0.38)	0.007
>54mg/d	10	984	−0.26(−0.45,−0.07)	
Total isoflavone dose				
≤14000mg	12	767	−0.09(−0.27,0.08)	0.30
>14000mg	11	1400	−0.02(−0.32,0.28)	
BMI				
≤25kg/m^2^	11	1137	0.05(−0.24,0.35)	0.07
>25kg/m^2^	9	760	−0.19(−0.40,0.01)	
Geographic regions				
North America	7	726	−0.04(−0.58,0.50)	0.04
South America	2	156	−0.23(−0.44,−0.01)	
Asia	3	224	0.23(−0.04,0.50)	
Australia	2	116	−0.18(−0.62,0.26)	
Europe	9	945	0.01(−0.34,0.36)	

1 SMD: standard mean difference

2 P value is from the meta-analysis of total effect of each subgroup, assessed by random-effect models

### Publication bias

**Figure 5:**
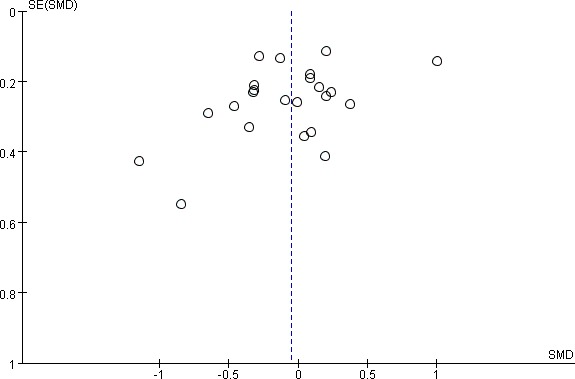
Funnel plot (with pseudo 95% CIs) of all individual studies in the meta-analysis Studies that evaluated the effect of oral isoflavone supplementation on endometrial thickness change were plotted with standard mean differences (SMDs) on the vertical axis and the SEs of the SMDs along the horizontal axis. Graph symbols were sized by weights

A statistical analysis of the Egger test and funnel plots were performed in all 23 studies. No significant publication bias was observed (Egger test, *P* = 0.624; Figure 5). Egger tests were also done in the subgroups, which also indicated no publication bias.

## DISCUSSION

To evaluate the effect of isoflavone supplement on endometrial thickness we performed this meta-analysis with 23 randomized controlled trials of oral isoflavone supplement intervention (totally 2167 subjects received isoflavone supplement or placebo). We found that isoflavone supplementation of more than 54mg per day could decrease endometrial thickness of 0.26mm in post-menopausal women. The subjects from North America had a significant decrease (−0.23mm) in endometrial thickness after isoflavone intervention, which was opposite to the response of Asian subjects (0.23mm increase). And no publication bias was observed.

Loss of estrogen could result in the increase of endometrial thickness [33]. Increase of endometrial thickness, especially in post-menopausal women, is an early pathologic feature and a predictor of endometrial cancer [34]. Dietary intake of phytoestrogens has been reported to protect women from estrogen-related diseases [35, 38]. Isoflavone mainly from soy beans, has shown a protective effect on endometrial cells and breast cells in animal studies [36]. Isoflavone has also shown a modification effect on gene promoter methylation [37]. Hypermethylation in gene promoter regions was linked to many diseases like cancer [39] and metabolism syndrome [40]. Genistein as a subclass of isoflavone was considered to be the most active compounds and has been reported to affect DNA methylation [41, 42].

The effect of isoflavone on endometrial cancer in women has also been investigated by many observational studies [43-46]. Zhang et al. performed a meta-analysis about soy intake and endometrial cancer risk and found that soy food intake was associated with lower endometrial cancer risk in 10 related observational studies [46]. They found the highest reported soy intake compared with the lowest reduced the risk of endometrial cancer by 19%. This finding shows that the intake of soy foods has an positive effect on human endometrial. Our results were in concordance with Zhang's results since endometrial thickening is a risk factor of endometrial cancer.

Our analysis suggested that isoflavone supplementation dose of more than 54mg per day might decrease endometrial thickness in post-menopausal women which were consistent with a cohort study. Ollberding et al. performed a prospective study followed up for an average of 13.6 years and observed an inverse association between dietary isoflavones intake and the risk of endometrial cancer [45]. Every 100g soybeans contains about 109mg isoflavone, this means if we want to gain more than 54mg isoflavone we need to consume more than 50g soybeans or soy production. Dietary soy isoflavone intake in older Japanese American women was reported as 10.2mg per day [47]. More soy or soy product was needed to produce a beneficial effect on endometrial thickness in post-menupausal women.

Our results also show that subjects from North America had a significant decrease in endometrial thickness after isoflavone intervention, which was opposite to the response of Asian subjects. This difference could be contributed by different genetic background and dietary patterns between populations. Asian diet is rich in isoflavone due to more soy and soy products intake [48], which might result to a higher background level of isoflavone in their body; and additional supplementation may not be beneficial. North American had less isoflavone intake from their regular diet and the supplementation might be more effective [49].

This meta-analysis has some advantages: due to the nature of the studies, randomized double-blind controlled trials provided the most solid evidence for the effect of isoflavone supplementation on endometrial thickeness. In addition, with 23 RTCs and over 2100 subjects, the results from this large study should be stable with large power. Furthermore, the endometrial thickness from all the included studies was measured by transvaginal ultrasounds. The method is the most precise method in the current age, which minimized the heterogeneity of measurement [50]. Some potential limitations should be addressed. First, though we used the change of endometrial thickness before and after supplementation, the technology of endometrial thickness measurement improvement through years might affect the results. Second, the endometrial thickness may be affected by internal hormone exposure. Though 21 over 23 studies included only menopause women for the study, the different time duration after menopause might affect the endometrial thickness and response to hormone, which increase the heterogeneity of the study. The equol producer phenotype is important as it can reflect gut metabolite of soy isoflavone *in vivo* [51]. In human, only 30%-50% of the population are capable of converting daidzein to equol and equol's biological activities differs from its parent compound [52, 53]. However, we could not get the information about equol producers in almost all the included studies. In addition, the sample size is still not large enough, which limited the power to explore modification effect.

In summary, with meta-analysis we found that isoflavone supplementation might produce different effects on populations and the daily dose of isoflavone supplementation maybe important to the results. Additional large and long follow-up studies should be performed to confirm our results and explore the exact mechanism of isoflavone's effect on endometriun.

## MATERIALS AND METHODS

### Search strategy and selection criteria

We conducted a systematic meta-analysis according to the QUORUM (Quality of Reporting of Meta-analyses) guidelines [54]. An electronic search was performed on the PubMed (http://www.ncbi.nlm.nih.gov/pubmed) (from 1950 up to August 2015), Embase (http://www.embase.com) (from 1966 up to August 2015), the Cochrane Library database (http://www.cochrane.org), web of science (http://www.webofknowledge.com), CINAHL (http://www.ebscohost.com), and WHO ICTRP (http://www.who.int/trialsearch) up to August 2015. Additional search was conducted according to reviews and reference lists of relevant articles. The relevant text word “isoflavone” paired with “endometrial” or “endometrium” was used for searching. The inclusion criteria were as following:

- Completed, published, randomized, and placebo-controlled trials
- With oral isoflavone, extractions of soy, or red clover as supplementation
- Participants must have been treated with isoflavone for over 3 months to avoid the acute effect of isoflavone on endometrial thickness
- Included participants were women of perimenopausal or postmenopausal with endometrial thickness measurement

The exclusion criteria were as following:

- Animal studies or *in vitro* studies.
- The language of the article is not English.
- Reviews, case-report, or comments.

Potentially relevant studies were collected in full text for further assessment of inclusion. All the papers were assessed for their relevance by two independent reviewers. And all the differences in data extraction were judged through discussion.

### Data extraction and quality assessment

Mean change and variance of endometrial thickness were collected at the latest time point. Data on participants' characteristics (menopausal status, mean age, country, BMI), type of intervention (isoflavone source, type of supplementation, dose), duration of intervention, methods used to measure endometrial thickness, and side-effect were also extracted from full articles. In the current study we got all the endometrial thickness data through the transvaginal ultrasounds detection. When the same intervention was published differently, data for analysis were extracted from the newest follow-up of the trails with longer duration of intervention. Authors were contacted for detail information like baseline endometrial thickness and the exact number of subjects who got endometrial thickness measurement etc.

The quality of the studies was assessed according to: concealment of treatment allocation, mention of randomization methods, appropriateness of randomization, mention of blinding methods, appropriateness of blinding, and whether the fate of all patients in the trial was known. A trial was scored one point for each area addressed, with a possible score ranging from 0 to 5 (highest level of quality) [55-57]

### Statistical analysis

All analysis were conducted with Stata software (version 12.0; Stata Corporation, College Station, TX) and REVMAN software (version 5.0; Cochrane Collaboration, Oxford, United Kingdom). The primary outcome was defined as the change in endometrial thickness between baseline and end of the trial. If the change of endometrial thickness was not reported in the study, we calculated it using the methods recommended in the Cochrane Handbook (http://handbook.cochrane.org/) for Systemic Review and Follman D's theory [58] for overview of clinical trials with continuous variables by assuming equal variance among trials [10-32].

Summary estimates of standard mean differences (SMDs) and 95% confidence intervals (95% CIs) were obtained from random-effect models [58]. Heterogeneity of treatment effects between studies was assessed using Cochran's test with *P* < 0.1 considered as statistical significant. The I^2^ statistic > 50% was considered heterogeneity significant between the trials [59]. We also conducted stratified analyses by the following factors to identify the sources of heterogeneity:

- age (= < 60y or > 60y)
- BMI (= < 25kg/m^2^ or > 25kg/m^2^)
- geographic region of subjects (north America, south America, Asia, Europe, Australia)
- daily isoflavone dose (in aglycone equivalents, = < 54mg/day, > 54mg/day) and total supplementation isoflavone dose (the total amount of isoflavone intake of the whole supplementation period, in aglycone equivalents, = < 14000mg, > 14000mg)

Potential publication bias was assessed with the Egger's test and represented graphically by Funnel plots [60].
